# A Novel Small Molecular Antibody, HER2-Nanobody, Inhibits Tumor Proliferation in HER2-Positive Breast Cancer Cells *In Vitro* and *In Vivo*

**DOI:** 10.3389/fonc.2021.669393

**Published:** 2021-05-12

**Authors:** Yan Yan, Xiao Cheng, Lin Li, Rumeng Zhang, Yong Zhu, Zhengsheng Wu, Keshuo Ding

**Affiliations:** ^1^ Department of Pathology, School of Basic Medicine, Anhui Medical University, Hefei, China; ^2^ Key Laboratory of Flexible Electronics (KLOFE), Institute of Advanced Materials (IAM), Nanjing Tech University (NanjingTech), Nanjing, China; ^3^ Department of Pathophysiology, School of Basic Medicine, Anhui Medical University, Hefei, China; ^4^ Department of Pathology, The First Affiliated Hospital of Anhui Medical University, Hefei, China

**Keywords:** HER2, nanobody, HER2-VHH, domain antibody, breast cancer

## Abstract

Breast cancer is the most common malignant cancer in women worldwide, especially in developing countries. Herceptin is a monoclonal antibody with an antitumor effect in HER2-positive breast cancer. However, the large molecular weight of Herceptin limited its employment. In this study, we constructed and screened HER2-nanobody and verified its tumor-suppressive effect in HER2-positive breast cancer cells. HER2-nanobody was established, filtrated, purified, and was demonstrated to inhibit cell total number, viability, colony formation and mitosis, and promote cell apoptosis in HER2-positive breast cancer cells *in vitro*. Treated with HER2-nanobody, tumor growth was significantly inhibited by both intratumor injection and tail intravenous injection *in vivo*. The phosphorylation of ERK and AKT was restrained by HER2-nanobody in HER2-positive breast cancer cells. RAS-RAF-MAPK and PI3K-AKT-mTOR are two important pathways involved in HER2. It was credible for HER2-nanobody to play the tumor suppressive role by inhibiting the phosphorylation of ERK and AKT. Therefore, HER2-nanobody could be employed as a small molecular antibody to suppress HER2-positive breast cancer.

## Introduction

The incidence of breast cancer ranks first in female malignancies worldwide (1, 2). According to epidemiological statistics in 2018, breast cancer tied for first with lung cancer in new tumors (11.6%), ranked first among female tumors (24.2%), and ranked fifth among deaths (6.6%). In Asia, the incidence of breast cancer in 2018 was 43.6%, the mortality was 49.6%, and the 5-year prevalence was 38.2% (3). The main treatments for breast cancer were surgery, radiotherapy, chemotherapy, hormonotherapy and bio-targeted therapy (4), but the curative effect is not satisfactory.

HER2 (Human Epidermal Growth Factor Receptor 2), a member of the epidermal growth factor receptor (EGFR) family, also known as ERBB2 or c-erbB2. HER2 was reported to promote cell proliferation *via* several downstream pathways including E-cadherin and ATF4 (5, 6). Overexpression of HER2 inhibited the anti-tumor immunity in the internal environment (7). In addition, HER2 was reported to relate with worse prognosis in many cancers including breast cancer and gastric cancer (8, 9). Herceptin was the first monoclonal antibody drug approved by the FDA (Food and Drug Administration) for breast cancer and gastric cancer patients with HER2 overexpression (10). Studies have shown that the combined use of Herceptin and chemotherapeutic drugs could effectively improve disease-free and overall survival in breast cancer patients (11–13). However, Herceptin was not effective for all patients and some patients would acquire drug resistance eventually (14, 15). Besides, cardiotoxicity was one of the side effects in Herceptin use (16). The development of novel drugs is necessary.

Heavy chain only antibodies, lacking light chains are naturally occurring in blood of camelidae as first reported in 1993 (17). Compared with the antigen binding fragment of conventional monoclonal antibodies, heavy chain only antibodies recognize their cognate antigen *via* the single variable domain of the heavy chain. This variable domain is also known as VHH (Variable domain of the heavy chain of a heavy chain-only antibody) or Nanobody. These Nanobodies have a molecular mass of 15,000 (18, 19). Nanobodies are suitable for prokaryotic expression and various eukaryotic expression systems (20, 21), and are widely used in the development of therapeutic antibody drugs, diagnostic reagents, affinity purification matrices, and scientific research, becoming an emerging force in a new generation of therapeutic biomedical and clinical diagnostic reagents (22–24). Previously, there were some reports about nanobodies targeting HER2: combining nanobodies targeting HER2 with photochemical internalization (PCI) on polymerized nanoparticles (NPs) that carry saponin achieved the selectivity of NPs (25); a combination of liposome and HER2-nanobody facilitated the localization of breast cancer cells by magnetic resonance imaging (MRI) (26); HER2-nanobody radiolabeled with 131I, 18F, or 117Lu pinpointed and evaluated HER2 protein expression and localization (27–29). There has even been a phase I study using ^68^Ga-labeled HER2-nanobody for PET/CT to evaluate HER2 expression in breast cancer (30). However, few reports were published about HER2-nanobodies with specific suppressive role on HER2 positive cancer cells. Establishing a tumor-suppressive HER2-nanobody will facilitate the development of HER2-targeting therapy in human cancers.

In this study, we successfully constructed a tumor-suppressive nanobody against HER2, and verified its function through cell functional and xenograft experiments. The HER2-nanobody (also described as HER2-VHH) was constructed through camel immunization, RNA extraction and amplification, phasmid (pMECS) ligation and prokaryotic expression. Through cell total number assay, MTT assay, cell colony formation assay and flow cytometry, the HER2-nanobody constructed was examined to suppress cell proliferation, mitosis and stimulate apoptosis in HER2-positive breast cancer cells. The HER2-nanobody suppressed the phosphorylation of ERK and AKT which was involved in the RAS-RAF-MAPK and PI3K-AKT-mTOR pathways, two important downstream signaling pathways of HER2 (31, 32). Moreover, the HER2-nanobody dramatically inhibited tumor growth of HER2-positive breast cancer cells *in vivo*. Therefore, the HER2-nanobody we constructed in this study had tumor suppressive effects in HER2-positive breast cancer cells. Agents based on this HER2-nanobody could be potentially used for HER2-positive breast cancer therapy.

## Materials and Methods

### Construction and Screening of HER2-Nanobody

#### Construction of HER2-Nanobody

**Figure 1:**
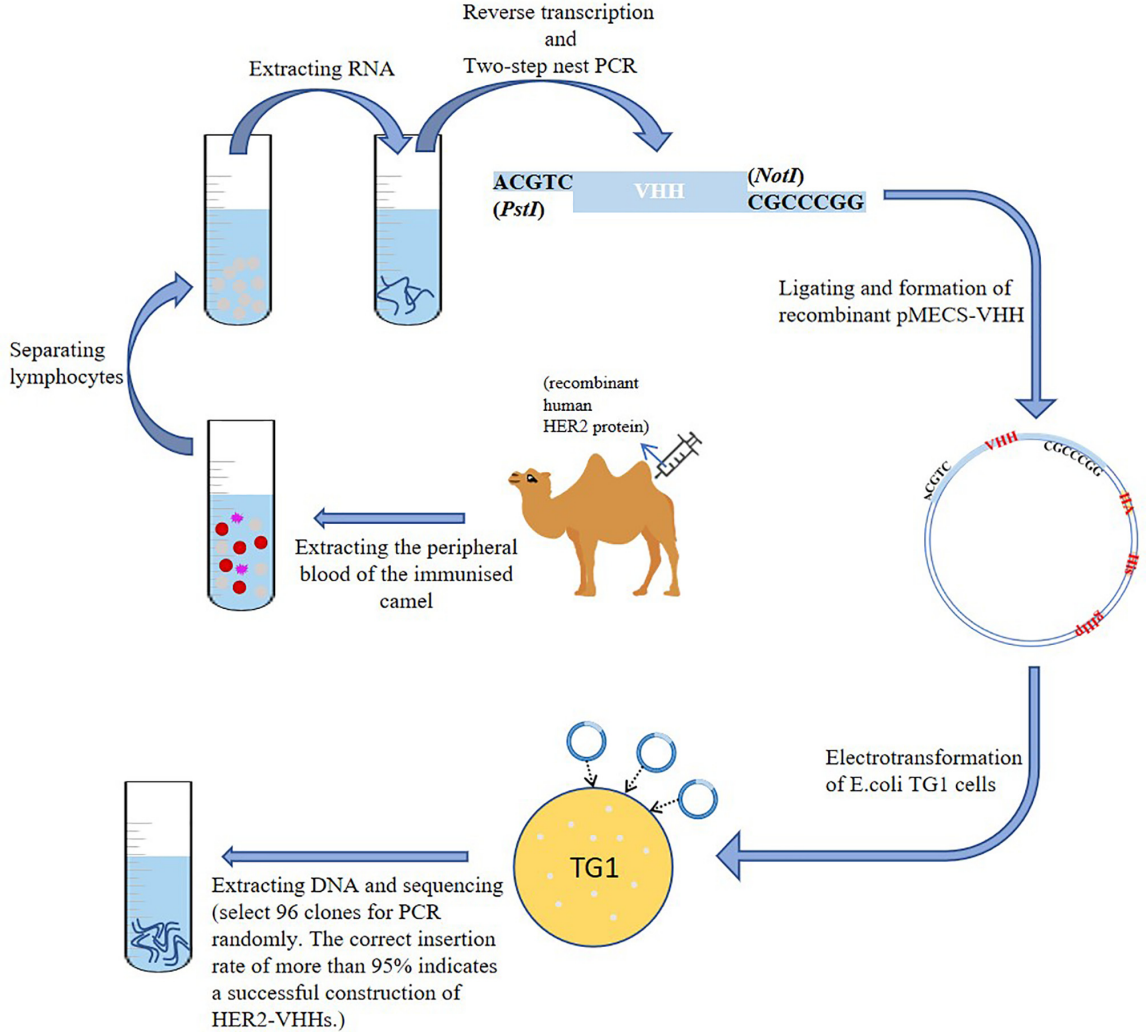
Construction of HER2-nanobody (HER2-VHH).

A schematic diagram of the construction of HER2-nanobody was shown in **Figure 1**. We mixed 1 mg of recombinant human HER2 protein (Beidamab, China) with an equal volume of freund’s adjuvant (filled up to 4 ml with PBS) (freund’s complete adjuvant for the first time, and freund’s incomplete adjuvant for the second time) (Sigma-Aldrich) and subcutaneously inject it into a male camel (4 site, 1 ml/site) (in camel breeding center of Jurong, Jiangsu, China) for 6 consecutive weeks. The peripheral blood of the immunized camel was extracted (300 ml) on the 45th day and situated at room temperature for 10 minutes. Density gradient centrifugation was used to separate lymphocytes from peripheral blood. The serum and blood cells were separated by centrifugation (3500 rpm, 10 min) and the blood cells were filled up with physiological saline to 300 ml. The diluted blood cells were mixed with 70% percoll (Sigma-Aldrich) and centrifuged (1500 rpm, 20 min). The separated lymphocytes were washed twice with equal volume of saline and added into 1 ml trizol (Invitrogen, USA). Reverse transcription was implemented as mentioned before to extract RNA and synthesize cDNA with oligo (dT) primer (33). Two-step nested PCR was carried out as mentioned previously (34). The primers of the first PCR used were: 5’-GTCCTGGCTGCTCTTCTACTTCC-3’, 5’-GGTACGTGCTGTTGAACTGTTCC-3’. The primers of the second PCR used were:5’-GATGTGCAGCTGCAGGAGTCTGGAGGAGG-3’, 5’-CTAGTGCGGC CGCTGAGGAGACGGTGACCTGGGT-3’ (35). The obtained VHH fragment was ligated with pMECS phagemid vector to form a pMECS/Nb recombinant (restriction endonuclease were: PstI-CTGCA^G; NotI-GC^GGCCGC). The obtained recombinant was electrotransformed into *E.coli* TG1 cells as mentioned previously (36). The size of the library reached 1.2×10^8^ individual transformants, selected 50 clones for PCR randomly. PCR was performed as recommended (36). The primers used were: 5’-CCGGAATTCCAGGTGCAGCTGGTGGAG-3’, 5’-CCCCTCGAGTCATGAGGAGACGGTGACCAT-3’.

#### Phage Display

**Figure 2:**
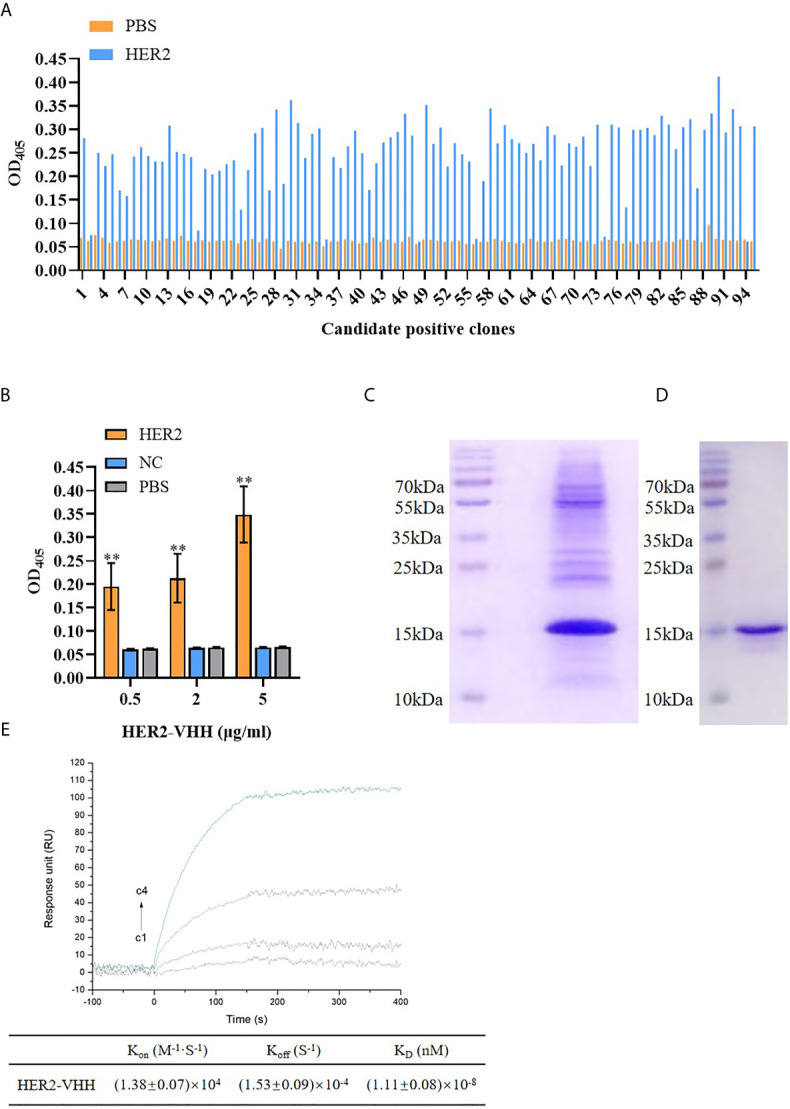
Screening of HER2-nanobody (HER2-VHH). **(A)**, HER2-VHH candidates were examined by ELISA assay. 100 μl/well 2 μg/ml HER2-VHHs were added to the titer plate coated with 10 μg/ml HER2 protein and incubated for 90 minutes. The HER2-VHH with the most content was screened. **(B)**, the binding of different concentration (0.5 μg/ml, 2 μg/ml, 5 μg/ml) of the screened HER2-VHH with HER2 protein were examined by ELISA assay (NC meant bovine serum albumin). For ELISA assay, OD405 was measured by a spectrophotometer. **(C)**, Ni-NTA spin columns affinity chromatography was performed to purify and verify the obtained HER2-VHH. **(D)**, gel filtration chromatography was performed for further verifying the obtained HER2-VHH. **(E)**, The K_D_ value between HER2 and the finally screened HER2-Nanobody was determined by the SPRi technology. HER2 dilutions were injected at concentrations of 2,10, 50, and 200 nM (C1–C4). The sensorgram represents the interaction of HER2 with the HER2-nanobody from the lowest concentration (lowest curve C4) to the highest concentration (top curve C1). **P < 0.01.

The HER2-nanobodies were selected by phage display. Four rounds of biopanning on 96-well plates were performed to enrich the phages expressing HER2-VHH specifically on the coat protein. Ninety-six single colonies randomly selected in each round were grown in TB medium. ELISA assay (described in *ELISA Assay*) was used to identify positive clones of HER2-nanobody. After sequencing, the identified positive clones were divided into different categories according to the CDR sequence. In our study, there were 68 positives among 96 clones. Sequencing analysis could be divided into 4 categories according to the CDR sequence (described in *Biacore* and **Figure 2E**). We selected antibodies with higher affinity for research. The plasmid was extracted from TG1 cells and further electrotransformed into *E.coli* WK6 cells. Cells were grown in TB medium. When the OD_600_ was between 0.6 and 0.9, 1 mM IPTG (isopropyl β-ɒ-1-thiogalactopyranoside) was used to shake overnight at 28°C to induce nanobody expression. The extract protein from the periplasm was purified by Ni-NTA spin columns affinity chromatography (described in *Ni-NTA Spin Columns Affinity Chromatography*) to purify HER2-nanobody. The purified HER2-nanobody was further analyzed by Gel filtration chromatography (described in *Gel Filtration Chromatography*).

#### ELISA Assay

ELISA assay was carried out to identify positive clones of HER2-nanobody and to detect the content of HER2-nanobody. Each well was coated with HER2 protein (10 μg/ml) overnight at 4°C and blocked with 0.5% BSA (BSA blocking solution) for 1 hour at room temperature. The candidate nanobody clones (2 μg/ml) were added to the experimental wells (100 μl/well) and incubated at room temperature for 90 minutes, the same amount of PBS was used as control. From the candidate nanobody clones, we screened the one with the strongest binding to HER2 protein and for further performed ELISA assay with different concentrations: 0.5 μg/ml, 2 μg/ml, 5 μg/ml. In detail, each well was coated with HER2 protein (10 μg/ml) (the same amount of PBS was used as control) overnight at 4°C and blocked with 0.5% BSA (BSA blocking solution) for 1 hour at room temperature. The candidate nanobody clones were added to the experimental wells (100 μl/well) (the same amount of NC (BSA, bovine serum albumin, Sigma, USA) was used as control) and incubated at room temperature for 90 minutes. We washed the wells with TBST for 5 times and added the secondary antibody (1:20000) to incubate at room temperature for 60 minutes. Finally, the ELISA plate was washed for 3 times with TBST and developing for 15 minutes in dark. The absorbance at 405 nm (OD405) was measured by a spectrophotometer (ThermoFisher).

#### Biacore

Biacore assay was carried out to detect the affinity of HER2-nanobody for HER2 protein according to the description in previous study (35). In short, after the HER2 ligand on the chip was fixed, HER2-nanobody incubation and chip regeneration were performed cyclically. K_D_ between HER2 protein and HER2 nanobody was determined by SPRi technique. HER2 dilutions were injected at concentrations of 2,10, 50, and 200 nM (C1–C4). The sensorgram represented the interaction of HER2 with the HER2-nanobody from the lowest concentration (lowest curve C1) to the highest concentration (top curve C4).

#### Ni-NTA Spin Columns Affinity Chromatography

Ni-NTA spin columns affinity chromatography was performed to purify the obtained HER2-nanobody. We washed NI-IDA with 0.05% TBST for 5 times and added 1 ml 0.5% BSA to block non-specific antigens (shaken on the reverse shaker for one hour). Then the blocked NI-IDA was washed for 3 times with TBST and loaded the columns. The HER2-nanobody was added into the HER2 columns (3 ml/min). The effluent liquid was collected and SDS-PAGE electrophoresis was performed.

#### Gel Filtration Chromatography

Gel filtration chromatography was implemented to further purify and identify the obtained HER2-nanobody. In short, we filled the gel filter column with gel slurry and the preliminary purified HER2-nanobody was added to the column (A Hiload 16/60 Superdex75 Column (GE Healthcare) was then preformed on an ÄKTA purifier 10 system. Gel filtration was run in 50 mM Tris-HCl, 150 mM NaCl, 1mM DTT at pH 7.4. The injection volume was 5 ml. The collections were concentrated by ultrafiltration). When the sample flowed to 1 mm above the gel bed, the outlet was closed and the effluent was collected and SDS-PAGE electrophoresis was performed.

#### SDS-PAGE Electrophoresis

The sample spotting and electrophoresis process were carried out according to the description in previous study (36). After electrophoresis, the gel was taken out and fixed with fixative at 4°C overnight. The next day, the gel was treated with staining solution for one hour and then washed with distilled water several times until a clear protein band was formed.

### Functional Experiments and Mechanism Exploration of HER2-Nanobody

#### Cell Lines and Cell Culture

Breast cancer cell line MCF-7 (barely expresses HER2), BT474 (strongly expresses HER2) and SKBR3(strongly expresses HER2) [obtained from ATCC (the American Type Culture Collection) (Rockville, MD)] were used in this study. The culture conditions were: RPMI 1640 medium [Invitrogen, USA, containing 10% fetal bovine serum (FBS, Invitrogen, USA)] and 5% CO_2_ at 37°C as recommended.

#### Cell Functional Assays

Cell total number assay, MTT assay and cell colony formation assay were implemented as mentioned before (36, 37). In cell total number assay, 1×10^5^ cells were seeded into 6-well dishes, 0, 10 or 20 μmol/L HER2-nanobody or NC-nanobody was added after 24 hours and cells were counted continuously for 5 days. In MTT assay, 2000 cells (MCF-7) or 5000 cells (BT474 and SKBR3) were seeded into 96-well dishes, 0, 2.5, 5, 10 or 20 μmol/L HER2-nanobody or NC-nanobody was added after 24 hours and cell viability (OD570nm) was detected after 72 hours. In cell colony formation assay, 1500 cells (MCF-7) or 2500 cells (BT474 and SKBR3) were seeded into 6-well dishes and 0, 10 or 20 μmol/L HER2-nanobody or NC-nanobody was added after 24 hours. Cell colony images were pictured and cell colony numbers were counted after 14 days.

#### Flow Cytometry

Cell apoptosis and cell cycle examinations were implemented by flow cytometry as mentioned before (33). Cells were treated with HER2-nanobody (0 μmol/L or 10 μmol/L) for 96 hours. Analysis of cell cycle experiments results (as shown in **Figures 4C, D**). The red part on the left side of the figure indicates that the cell is in the G1 phase of cell division, which refers to the gap time before DNA replication is completed from mitosis; the part framed with diagonal lines in the middle indicates that the cell is in the S phase of cell division, which refers to the DNA replication phase; The red part on the right indicates that the cell is in the G2 phase of cell division, which refers to the period of time from the completion of DNA replication to the beginning of mitosis.

#### Western Blot

Protein levels including ERK, p-ERK, AKT, p-AKT and control β-actin were examined by western blot as mentioned before (36). Mouse monoclonal antibody against β-actin (1:5000, Sigma, USA) and rabbit polyclonal antibodies against ERK (1/2) (1:1000), p-ERK (1/2) (1:3000), AKT (1:1000) or p-AKT (1:3000) (all from Proteintech Group, USA) were employed. The specific schedule was as follows: transmembrane time was 90 minutes and transmembrane current was 300 mA; blocking time was 45 minutes; primary antibody incubation time was 2 hours; secondary antibody incubation time was 1 hour.

### Xenograft Analysis

#### Xenograft Analysis

The animal-related experiments in this study were conducted in accordance with the guidelines of the Institutional Animal Care and Use Committee (available from www.iacuc.org). The work has been approved by the Institutional Animal Care and Ethics Committee of Anhui Medical University. All mice were raised in SPF (Specific Pathogen Free)-class housing of laboratory. Thirty 4-week female BALB/c-nu/nu mice (GemPharmatech Co, Ltd, Jiangsu, China) were unilaterally injected into mammary fat pad with HER2-positive breast cancer cells SKBR3 (5×10^6^/125 μl/site) and 24 of the 30 mice formed palpable tumors. After 15 days, tumors were about 200 mm^3^, these mice were divided into two groups randomly (12 mice in each group): the mice in group1 were injected intratumorally with HER2-nanobody (20 mg/kg) (8 mice) or the same amount of PBS (4 mice) once a week; the mice in group2 were injected tail-intravenously with HER2-nanobody (20 mg/kg) (8 mice) or the same amount of PBS (4 mice) once a week. Tumor volumes (V (mm^3^) = L × W2 × Π/6) were measured every four days and the mice were executed 39 days after cell injection. Tumors were isolated, pictured, and then fixed with 4% formalin and made into paraffin section.

#### Immunohistochemistry and TUNEL

Positivity of ki-67 and apoptotic nuclei in tumors formed by SKBR3 cells were detected by Immunohistochemistry (IHC) and terminal transferase deoxyuridine triphosphate nick end labeling (TUNEL) respectively as mentioned before (37, 38). The UltraSensitive-SP kit (Maxin-Bio, Fuzhou, China) and mouse polyclonal antibody against ki-67 (Zhongshan Goldenbridge Biotechnology Co, Beijing, China) was used. The staining results were evaluated independently by two senior pathologists in the Pathology Department of the First Affiliated Hospital of Anhui Medical University.

### Statistical Analyses

In this study, three or more repeated experiments were averaged to represent the final results. Unpaired two-tailed t test was employed in PCR, ELISA assay, cell total number assay, MTT assay, cell colony formation assay, flow cytometry and xenograft analyses. Pearson’s chisquare test was employed in immunohistochemistry. *P*<0.05 was considered statistically significant.

## Results

### Construction of HER2-Nanobody

A schematic diagram of the construction of HER2-nanobody was shown in **Figure 1**. We immunized camels with HER2 protein and extracted peripheral blood after eliciting the immune response. Lymphocytes were isolated by density gradient centrifugation. The total RNA of the immunized lymphocytes was extracted and the target fragments were obtained and amplified by reverse transcription and two-step nest PCR. The fragments of the preliminary HER2-nanobody were ligated to pMECS to form a recombinant phagemid (pMECS/Nb) and finally transferred it into *E.coli* TG1 cells for amplification. The details were described in the *Materials and Methods* section. In our library, the size was 1.2×10^8^. After each round of panning we randomly chose 96 clones for screening in ELISA.

### Screening of HER2-Nanobody

The amount of HER2-specific nanobody in the extract and the antigen-specificity of the Nb was assessed by ELISA. As expounded in **Figure 2A**, The amount of HER2-nanobodies were significantly higher than those of PBS control respectively. The most content of HER2-nanobody was selected for further study. As shown in **Figure 2B**, the HER2-nanobody showed a dramatically stronger binding to HER2 protein compared with NC or PBS control in different concentrations (0.5 μg/ml, 2 μg/ml, 5 μg/ml). The obtained HER2-nanobody was further verified and purified by Ni-NTA spin columns affinity (**Figure 2C**) and gel filtration chromatography (**Figure 2D**). Compared with the traditional HER2 antibody (185kDa) and trastuzumab (298kDa), the molecular weight of HER2-nanobody that we finally screened was only about 15kDa. Moreover, The K_D_ value between HER2 and the finally screened HER2-nanobody was determined by the SPRi technology. K_D_=(1.11 ± 0.08)×10^-8^, Kon=(1.38 ± 0.07)×10^4^, Koff=(1.53 ± 0.09)×10^-4^ (**Figure 2E**).

### HER2-Nanobody Restrained Cell Proliferation in HER2-Positive Breast Cancer Cells

**Figure 3:**
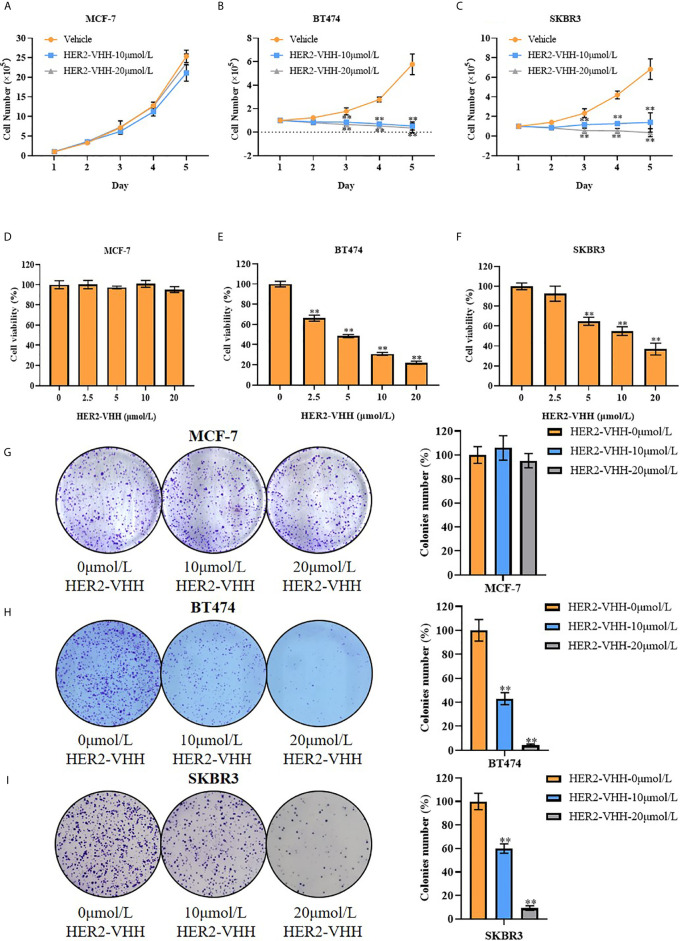
HER2-nanobody (HER2-VHH) restrained cell proliferation in HER2-positive breast cancer cells. **(A–C)**, cell total number assay, 1×10^5^ cells were seeded into 6-well dishes, 0, 10 or 20 μmol/L HER2-VHHs were added after 24 hours and cells were counted continuously for 5 days. **(D–F)**, MTT assay, 2000 cells (MCF-7) or 5000 cells (BT474 and SKBR3) were seeded in 96-well dishes, 0, 2.5, 5, 10 or 20 μmol/L HER2-VHHs were added after 24 hours and cell viability (OD570nm) was detected after 72 hours. **(G–I)**, cell colony formation assay, 1500 cells (MCF-7) or 2500 cells (BT474 and SKBR3) were seeded in 6-well dishes and 0, 10 or 20 μmol/L HER2-VHHs were added after 24 hours. Cell colony images were pictured and cell colony numbers were counted after 14 days. **P < 0.01.

Breast cancer cell line MCF-7 (barely expresses HER2), BT474 (strongly expresses HER2) and SKBR3 (strongly expresses HER2) were cultured and treated with different concentrations of HER2-nanobody [the following were designated as MCF-7/BT474/SKBR3-HER2-VHH (-treatment concentration)] or Negative control nanobody (HSA-nanobody, NC-VHH). Cell total number assay, MTT assay and cell colony formation assay were implemented to detect cell proliferation and viability. As expounded in **Figures 3A–C**, cell total numbers of both BT474 and SKBR3 cells were memorably reduced when treated with 10 μmol/L or 20 μmol/L HER2-nanobody compared with vehicle control within 5 days, while there was no significant change in MCF-7. After treated with concentration gradient of HER2-nanobody (0, 2.5, 5, 10 or 20 μmol/L) for 3 days, the cellular viabilities of MCF-7 didn’t change obviously (**Figure 3D**). BT474 and SKBR3 cells showed significantly decreased cell viabilities compared with control respectively (**Figures 3E, F**). Congruously, the capacity of cell colony formation in both BT474-HER2-VHH (10 μmol/L or 20 μmol/L) and SKBR3-HER2-VHH (10 μmol/L or 20 μmol/L) cells faded compared with control group and the trend was more obvious in higher HER2-nanobody concentration (20 μmol/L), while there was no significant change in MCF-7 when treated with different concentration of HER2-VHH (**Figures 3G–I**). Meanwhile, NC-VHH showed no significant effect on the proliferation of BT474 cells (**Figure S1**). Therefore, HER2-nanobody restrained cell proliferation in HER2-positive breast cancer cells.

### HER2-Nanobody Accelerated Cell Apoptosis and Restrained Cell Karyokinesis in HER2-Positive Breast Cancer Cells

**Figure 4:**
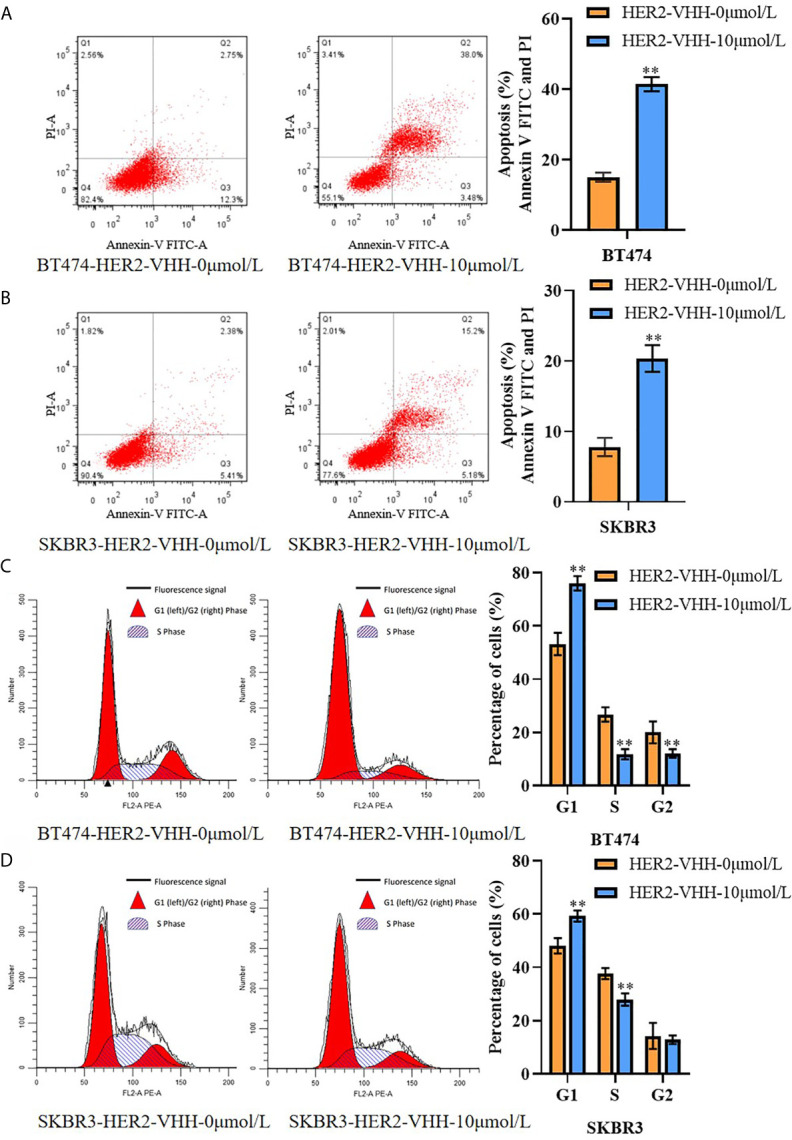
HER2-nanobody (HER2-VHH) accelerated cell apoptosis and restrained cell karyokinesis in HER2-positive breast cancer cells. BT474 and SKBR3 cells were treated with HER2-VHH (0 μmol/L or 10 μmol/L) for 96 hours. **(A, B)**, cell apoptosis was implemented by flow cytometric analyzation. **(C, D)**, cell cycle analysis of cancer cells was implemented by flow cytometric analyzation. **P<0.01.

We next performed flow cytometry to explore the influence of HER2-nanobody on cell apoptosis and karyokinesis in HER2-positive breast cancer cells. As expounded in **Figures 4A, B**, the proportion of apoptotic cells in BT474-HER2-VHH-10μmol/L (41.48%) and SKBR3-HER2-VHH-10μmol/L (20.38%) increased relatively compared with BT474-HER2-VHH-0μmol/L (15.05%) and SKBR3-HER2-VHH-0μmol/L (7.72%). Meanwhile, the proportion of cells in G1 phase increased from 53.25% (BT474-HER2-VHH-0μmol/L) and 48.08% (SKBR3-HER2-VHH-0μmol/L) to 76.06% (BT474-HER2-VHH-10μmol/L) and 59.28% (SKBR3-HER2-VHH-10μmol/L), the proportion of cells in G2 phase decreased from 20.02% (BT474-HER2-VHH-0μmol/L) and 14.24% (SKBR3-HER2-VHH-0μmol/L) to 12.11% (BT474-HER2-VHH-10μmol/L) and 12.80% (SKBR3-HER2-VHH-10μmol/L), and the proportion of cells in S phase decreased from 26.74% (BT474-HER2-VHH-0μmol/L) and 37.68% (SKBR3-HER2-VHH-0μmol/L) to 11.83% (BT474-HER2-VHH-10μmol/L) and 27.93% (SKBR3-HER2-VHH-10μmol/L) respectively (**Figures 4C, D**). These results showed that the proportion of cells in G1 phase increased and that in S and G2 phase decreased memorably after treating with 10 μmol/L HER2-nanobody in both BT474 and SKBR3 cells, indicating that more cells stayed in resting state and fewer cells in mitotic phase. Therefore, HER2-nanobody accelerated cell apoptosis and restrained cell mitosis in HER2-positive breast cancer cells.

### HER2-Nanobody Inhibited Phosphorylation of ERK and AKT in HER2-Positive Breast Cancer Cells

**Figure 5:**
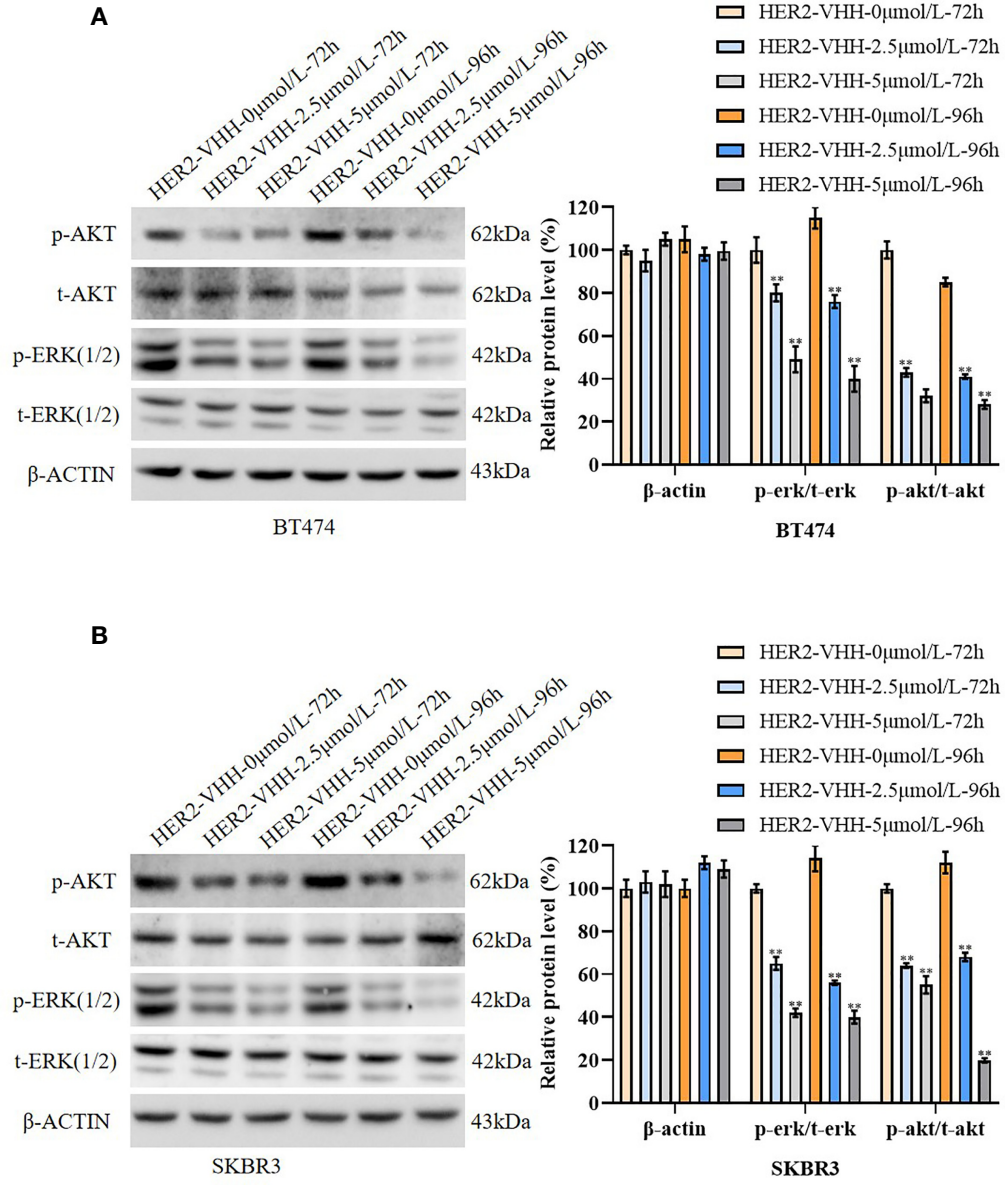
HER2-nanobody (HER2-VHH) inhibited phosphorylation of ERK and AKT in HER2-positive breast cancer cells. **(A, B)**, BT474 and SKBR3 cells were treated with 0, 2.5 or 5 μmol/L HER2-VHH for 72 or 96 hours. Protein levels of p-AKT, t-AKT, p-ERK (1/2), t-ERK (1/2) and house-keeping protein β-actin were examined by western blot. The quantitative protein levels were showed on the right respectively. **P<0.01.

To explore the inhibitory mechanism of HER2-nanobody on HER2-positive breast cancer cells, the protein levels of t-ERK (1/2), p-ERK (1/2), t-AKT and p-AKT were examined by western blot. The results were graphed from three blots and each protein levels were statistically compared to the HER2-VHH-0μmol/L-72h group respectively. As expounded in **Figures 5A, B**, the protein levels of p-ERK (1/2) and p-AKT decreased memorably in BT474 and SKBR3 cells after treated with 2.5μmol/L and 5μmol/L HER2-nanobody compared with control, while the protein levels of t-ERK (1/2) and t-AKT didn’t change. Moreover, higher concentration of HER2-nanobody (5 μmol/L) and/or longer treatment time (96 hours) induced the phosphorylation of ERK (1/2) and AKT to reduce more remarkably. Therefore, HER2-nanobody inhibited phosphorylation of ERK and AKT in HER2-positive breast cancer cells.

### HER2-Nanobody Inhibited Tumor Growth of HER2-Positive Breast Cancer *In Vivo*

**Figure 6:**
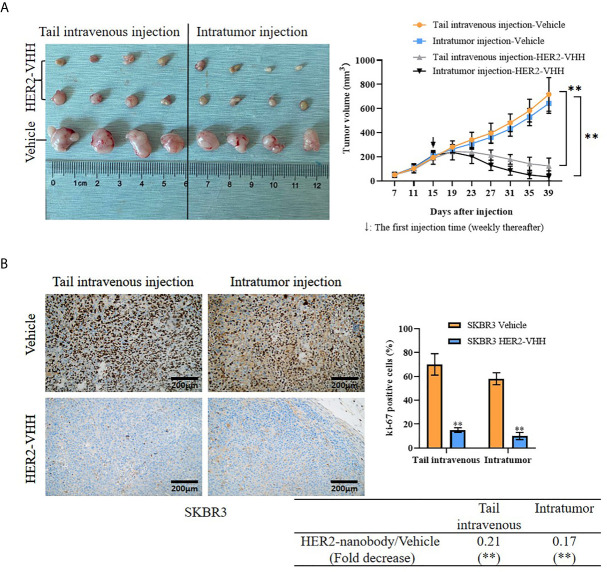
HER2-nanobody (HER2-VHH) inhibited tumor growth of HER2-positive breast cancer *in vivo*. SKBR3 cells (5×10^6^/125 μl/site) were unilaterally injected into mammary fat pad of 4-week-old female BALB/c-nu/nu mice. After 15 days when palpable tumors were about 200 mm^3^, the mice were received HER2-VHH (20 mg/kg) or Vehicle control (PBS) intratumorally or tail-intravenously once a week (↓: The first injection time). Thirty-nine days after cell injection, the mice were executed and tumors were isolated. **(A)**, physical map of tumor tissues (left) and tumor growth curves (right). **(B)**, the positivity of ki-67 in the tumor sections was detected by immunohistochemistry. **P<0.01.

Moreover, we examined the role of HER2-nanobody on HER2-positive breast cancer cells *in vivo*. Thirty 4-week female BALB/c-nu/nu mice were unilaterally injected into mammary fat pad with HER2-positive breast cancer cells SKBR3 (5×10^6^/125 μl/site) and 24 of the 30 mice formed palpable tumors after 15 days (about 200 mm^3^). The mice were treated randomly with HER2-nanobody (20 mg/kg) or PBS as control intratumorally or tail-intravenously. Tumor sizes were measured every 4 days and the mice were executed 39 days after cell injection. As expounded in **Figure 6A**, the tumor size in the HER2-nanobody injection groups (both intratumorally and tail-intravenously, especially the intratumoral-injection group) were much smaller compared with the PBS injection control group. In our present condition, we did not observe off-target toxicity in the *in vivo* model. In addition, immunohistochemistry was carried out to detect ki-67 levels in the tumor sections to determine cell proliferation and TUNEL assay was carried out to determine cell apoptosis in the tumor sections. Concordantly, tumors in mice treated with HER2-nanobody showed significantly lower levels of ki-67 compared with control in both intratumoral-injection group and tail-intravenous-injection group. The fold decrease of ki-67 positivity (HER2-nanobody/vehicle) was 0.21 (*P*<0.01) in tail-intravenous-injection group and was 0.17 (*P*<0.01) in intratumoral-injection group (**Figure 6B**). On the other hand, tumors in mice treated with HER2-nanobody showed significantly higher apoptotic nuclei compared with control in both intratumoral-injection group and tail-intravenous-injection group. The fold decrease of apoptotic nuclei (HER2-nanobody/vehicle) was 7.69 (*P*<0.01) in tail-intravenous-injection group and was 9.01 (*P*<0.01) in intratumoral-injection group (**Figure S2**). Therefore, HER2-nanobody also inhibited tumor growth of HER2-positive breast cancer *in vivo*.

## Discussion

Surgery combined with radiotherapy, chemotherapy, endocrine therapy and/or targeted therapy is a globally recognized therapy for breast cancer (4). However, multiple recurrences after surgery and multiple complications after radiotherapy and chemotherapy require us to further explore superior treatments. Nanobody is the smallest molecular binding to target antigen (38–40) and several kinds of nanobodies were reported to be potential agents that could be used in tumor therapy. For example, nanobody based CAR (chimeric antigen receptor) -T cell could better target the tumor microenvironment and inhibit solid tumor growth of melanoma (41); EGFR-nanobody produced by DNA immunization could effectively inhibit EGFR signaling and inhibit the growth of head and neck squamous cell carcinoma (42, 43); The production of CD38-nanobody provided new ideas for the treatment of multiple myeloma (44); Nanobodies targeting CEA could be quickly absorbed by non-small cell lung cancer and targeted specific receptors (45). In this study, we have successfully constructed a HER2-nanobody that could directly suppress tumor proliferation in HER2-positive breast cancer.

HER2 is closely related to the occurrence and development of human breast cancer (9, 46–49). Approximately 15% -20% of breast cancer patients showed HER2 overexpression (50). Herceptin, a HER2-targeted drug, is widely used in the treatment of HER2-positive breast cancer and metastatic breast cancer (10). Herceptin combined with paclitaxel and docetaxel has been testified to reduce mortality and improve prognosis in HER2-positive breast cancer patients (51–53). Irreversible cardiotoxicity, however, especially congestive heart failure, was a non-negligible adverse effect after Herceptin administration (16). Herceptin has also been reported to disrupt the function of mitochondria in cardiomyocytes (54). The invention of a more effective targeted drug with fewer side effects and easier access is imminent. Herein, we have successfully constructed a nanobody targeting HER2 with smaller molecular weight for HER2-positive breast cancer inhibition. The obtained HER2-nanobody was verified and purified by Ni-NTA spin columns affinity chromatography and gel filtration chromatography. Compared with the traditional HER2 antibody (185kDa) and targeted drug Herceptin (298kDa), the molecular weight of the HER2-nanobody was only about 15kDa. Cell total number assay, MTT assay, and cell colony formation assay showed that the HER2-nanobody inhibited cell proliferation in HER2-positive breast cancer cells. HER2-nanobody increased the proportion of apoptotic cells in both BT474 and SKBR3 cells. HER2-nanobody also increased the G1 phase cells, decreased the S and G2 phase cells in BT474 and SKBR3. Moreover, HER2-nanobody inhibited the growth of solid tumors *in vivo*. Keyaerts, M. et al. reported that nanobody labeled with 68Ga-HER2 could be used as a benign and safe probe in PET/CT molecular imaging (30). Nikkhoi, S. K. et al. found that nanobodies against HER2 protein covalently coupled to liposomes might become a new weapon for targeting HER2-positive breast cancer (55). In addition, fusion of single-domain antibody against HER2 with human IgG1 Fc had been reported to inhibit the proliferation of HER2-positive breast cancer cells *in vitro* and *in vivo* (56). The nanobodies targeting HER2 were important tools to carry small molecular drugs or labeled molecules used for antigen localization or tumor suppressing. However, these previous reported HER2-nanobodies could not suppress HER2 positive cancer cells directly. A HER2-nanobody with direct tumor suppressive role could be better used for HER2 positive cancer therapy. We herein have successfully constructed a tumor suppressive HER2 nanobody that inhibited HER2-positive breast cancer both *in vitro* and *in vivo*. Therefore, agents based on this HER2-nanobody constructed in this study could be better potentially used for HER2 positive cancer therapy.

RAS-RAF-MAPK and PI3K-AKT-mTOR pathways are two important signaling pathways involved in HER2 (31, 32). ERK, also known as mitogen-activated protein kinase 1 (MAPK1), encodes a member of the MAP kinase family. ERK includes ERK1 (44kDa) and ERK2 (42kDa). In the RAS-RAF-MAPK pathway, upstream signaling molecules activate SDS, and the activated SDS binds to the RAS protein and further binds to GTP. The SDS-RAS-GTP complex activates MAPKKK, MAPKK, and MAPK (ERK) in turn. Phosphorylated ERK is then transported into the nucleus, producing a biological effect (57). AKT, also known as AKT serine/threonine kinase 1, encodes serine-threonine protein kinase. In the PI3K-AKT-mTOR pathway, upstream signaling molecules activate PI3K, which converts PIP2 to PIP3. PIP3 binds to the intracellular signaling protein PDK1 to phosphorylate AKT. Phosphorylated AKT further activates mTOR, thereby activating protein translation, promoting cell proliferation (58). HER2 is the most effective activator of the RAS-RAF-MAPK and PI3K-AKT-mTOR pathway (59, 60). In this study, p-ERK and p-AKT were found to be specifically reduced by HER2-nanobody. Therefore, the inhibitory effect of HER2-nanobody on the proliferation and pro-apoptosis in HER2-positive breast cancer cells might be achieved by inhibiting the phosphorylation of ERK and AKT.

In summary, a specific HER2-nanobody was successfully constructed, screened and verified to inhibit proliferation, promote apoptosis and suppress mitosis in HER2-positive breast cancer cells both *in vitro* and *in vivo*. HER2-nanobody suppressed the downstream RAS-RAF-MAPK and PI3K-AKT-mTOR pathways. Drugs derived from this HER2-nanobody could be potential treatment methods for HER2-positive breast cancer.

## Data Availability Statement

The original contributions presented in the study are included in the article/**Supplementary Material**. Further inquiries can be directed to the corresponding authors.

## Ethics Statement

The animal study was reviewed and approved by the Institutional Animal Care and Ethics Committee of Anhui Medical University.

## Author Contributions

YY constructed and screened HER2-nanobody and participated in xenograft analysis. XC performed cell culture, cell functional experiments, and xenograft analysis. LL performed Western blot. RZ participated in cell functional experiments. YZ performed Immunohistochemistry. ZW participated in data analysis and manuscript revision. KD participated in study design and writing. All authors contributed to the article and approved the submitted version.

## Funding

This work was supported in part by grants from the Natural Science Foundation of Anhui Province (2008085QH378, 2008085MH276) and National Nature Science Foundation of China (81972472).

## Conflict of Interest

The authors declare that the research was conducted in the absence of any commercial or financial relationships that could be construed as a potential conflict of interest.
