# Supplementary figures and images for: A Novel Small Molecular Antibody, HER2-Nanobody, Inhibits Tumor Proliferation in HER2-Positive Breast Cancer Cells In Vitro and In Vivo
Source: Front Oncol. 2021 May 12;11:669393. doi: 10.3389/fonc.2021.669393 (PMC8149955; doi:10.3389/fonc.2021.669393)

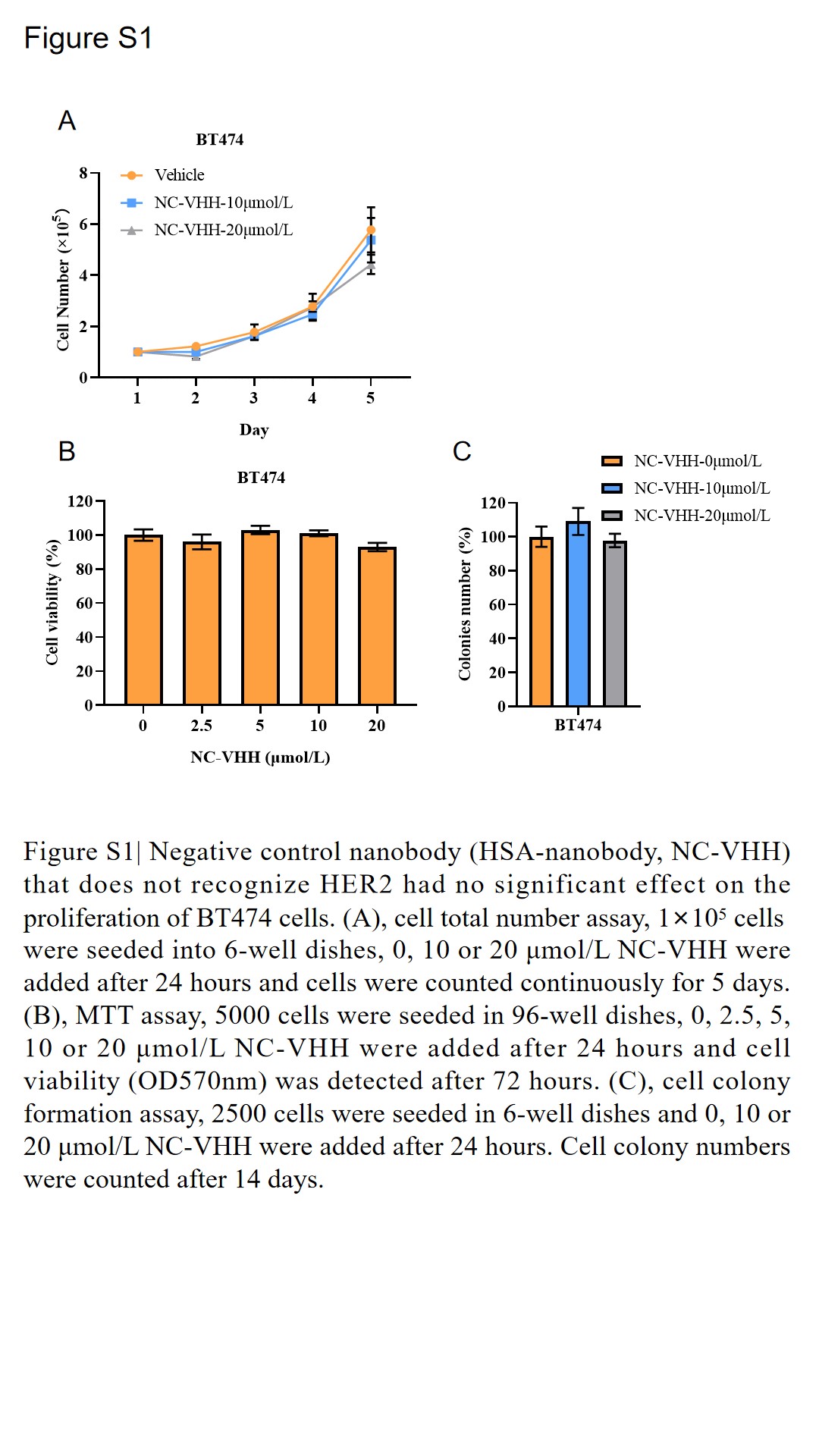

Supplement: Supplementary file 1 [file Image_1.jpg]

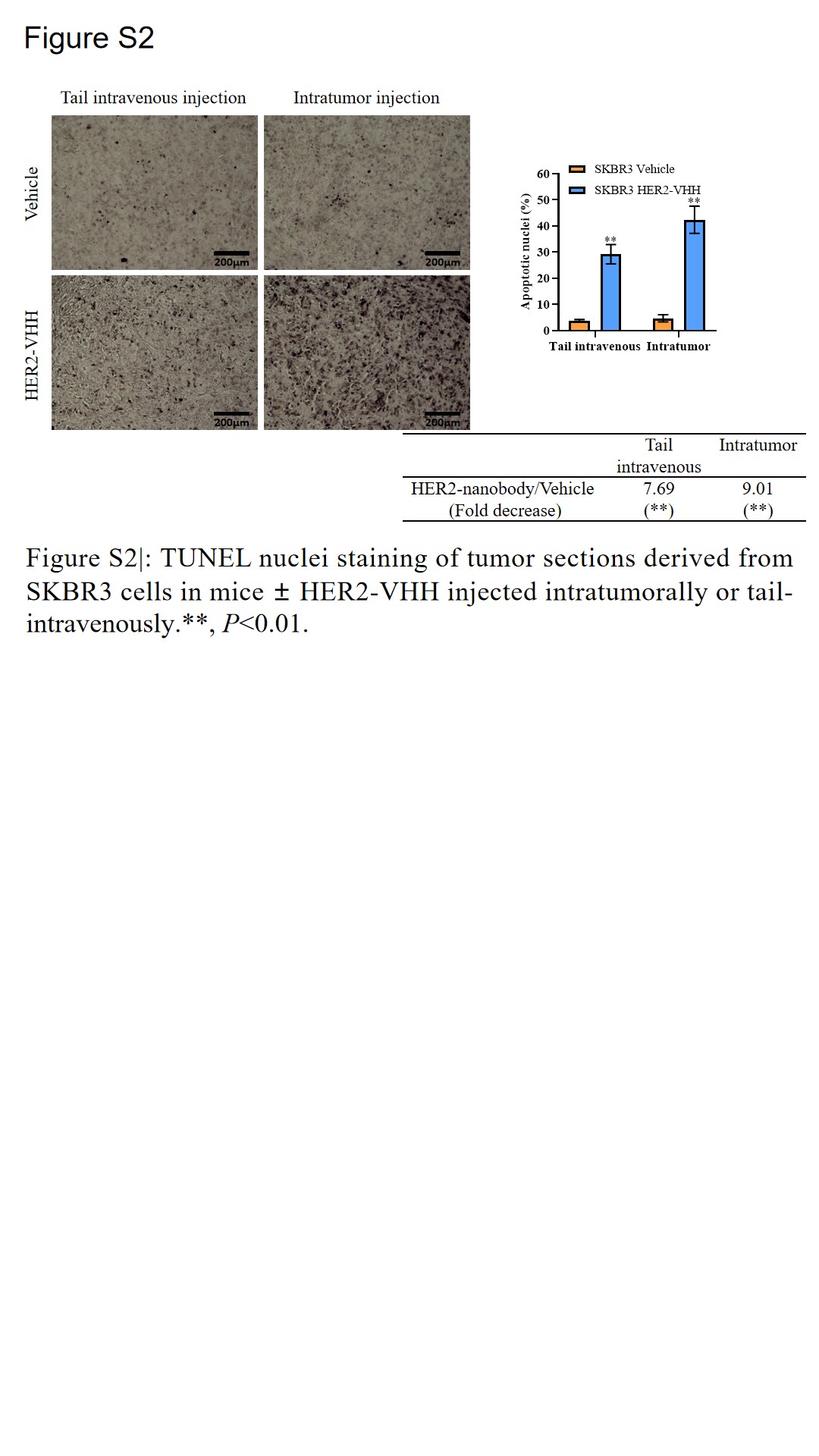

Supplement: Supplementary file 2 [file Image_2.jpeg]

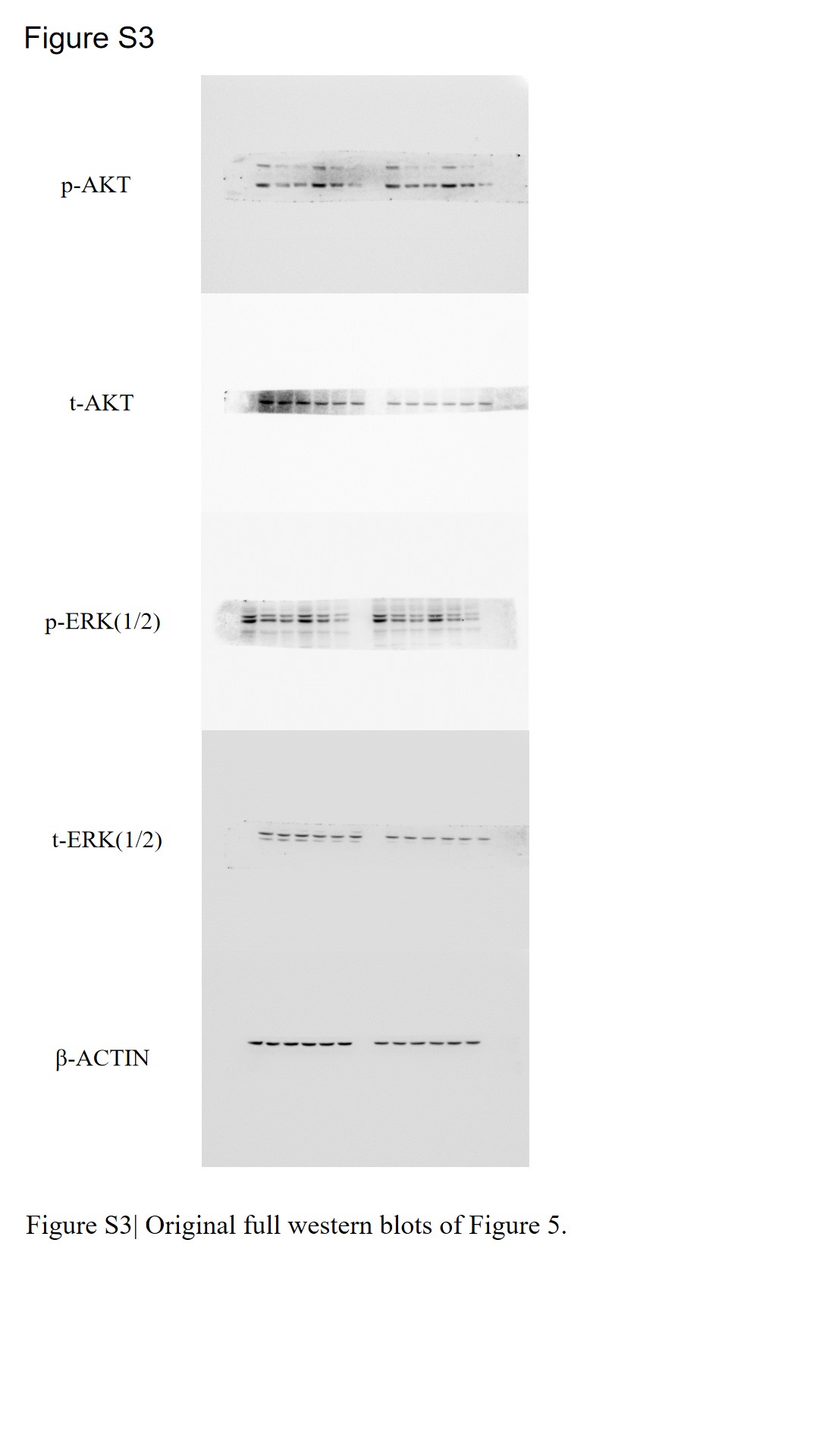

Supplement: Supplementary file 3 [file Image_3.jpeg]
